# Delivering bad news in clinical hematology: a personal perspective

**DOI:** 10.46989/001c.126214

**Published:** 2024-12-21

**Authors:** Mohamad Mohty, Finn Bo Petersen, Didier Blaise

**Affiliations:** 1 Sorbonne University, INSERM UMRs 938, Service d’Hématologie Clinique et Thérapie Cellulaire, Paris, France; 2 International Academy for Clinical Hematology, London, United-Kingdom; 3 Department of Hematology, Institut Paoli-Calmettes, Cancer Sports Management Lab, Aix- Marseille University, Marseille, France

**Keywords:** Delivering bad news, hematology, communication, patient support, cancer care, palliative care

Conversations that require delivering bad news to patients and families in clinical hematology often involves the disclosure of the patient having a cancerous diagnosis. These diagnoses include various forms of leukemia, lymphoma, plasma cell disorders, and myelodysplastic syndromes, all associated with significant morbidity and mortality from the disease itself as well as its treatment. The complex nature of these diseases requires clinicians to approach such conversations with empathy and clarity, making sure that the patient and family members understand the diagnosis and prognosis with and without treatment, while at the same time feeling supported by the hematologist. Thus, delivering information of this nature is a critical, yet challenging, element of clinical hematology practice, requiring a mixture of expertise, prepared communication, and understanding the patient’s social situation all providing the basis for an empathetic engagement. As every patient’s situation is unique, there is no written ‘recipe’ that can be used for all, with the challenge for the clinical hematologist to differentiate between different patient situations and individualize the conversation to the extent possible. The key to this in a way that is most helpful to the individual patient is for the clinical hematologist to be able to take time to prepare for the conversation as well as whatever time it takes to have the conversation with patient and family. Without the ability to have this time can result in patient confusion, increased anxiety, loss of trust with the clinician and ultimately, a lack of patient-centered care.^1^ This editorial aims to share our own practice and experience in communicating bad news to hematology patients with preparation, empathetic honesty, and a plan for mitigating the consequences of the bad news.

When delivering bad news, having time to properly prepare is crucial, notably because of the usual daily busy schedule is likely to make. Once the diagnosis is made, clinicians should gather relevant details about the diagnosis, prognosis, and treatment options, but also the patient’s personal history in order to provide an informed, honest, and tailored discussion. If meeting with the patient for the first time, this can be done by reading prior clinical notes, especially if from social workers, case workers and nurses. Creating a private, non-intrusive and uninterrupted conducive environment with sufficient scheduled time is essential and respects the gravity of the conversation and aids the patient’s ability to process information without distraction.^2^ This can be done in a small conference room with comfortable chairs, with it being important that the patient and clinician are at eye level and able to communicate at a normal voice level. At least one family member or trusted friend should be encouraged to be present to provide a second set of ears and support of the patient, however, if the patient insists to do the conversation alone after hearing this recommendation, it should be respected and not further belabored. An associate staff member in the form of a nurse, social worker, advanced practice provider or similar should be present as well, as well as taking notes for use in after visit summary the patient can be given as soon as the meeting finishes.

At the first meeting, understanding a patient’s current knowledge of their condition sets the foundation for effective communication. Beginning with asking the patient ‘what is your understanding of why Dr. X wanted you to have this meeting’ is an open-ended question which allows clinicians to assess the patient’s baseline understanding of their situation and provides the first tool for providing a personalize the approach. This step aligns with patient-centered care principles, fostering an environment where patients feel heard and understood.^3^ The language used in the discussions should be both clear and compassionate, if possible and appropriate, modulated according to the patient’s educational and/or ‘medical sophistication’ level, but as a rule of thumb avoiding technical jargon that may confuse or overwhelm. This includes when the patient is a medical professional. Assuming that their medical education makes them understand the common acronyms and technical details of hematology is not recommended, as with today’s specialization, we find that physicians who are not hematological oncologists are as bewildered about our field as are patients with no medical education. Honest and as precise laymen language helps convey the reality of the situation, while empathy can be expressed through phrases such as “I’m really sorry to have to tell you this, but unfortunately we have to deal with the reality that etc.”.^1^ Sitting while speaking, showing an open posture, and maintaining eye contact are tools of body language important in conveying empathy.^4^ Studies indicate that compassion in communication mitigates patient distress and builds trust, contributing to better coping outcomes.^5^ Also, it is important to gauge how much information the patient and family wish to receive, while delivering the news with clarity and empathy. It is of the utmost importance to address and validate the patient’s emotional reactions and outline the next steps and summarize key points to ensure patient understanding.^1^ It goes without saying, that the clinician leading the conversation should not sit behind a computer monitor of any kind, or have any distractions like a phone or pager (and arguably a wristwatch), If the preparation allows the clinician to conduct the conversation without having to refer to printouts or other written information, the better this allows the clinician to focus on the patient and the patient’s absorption and processing of the information.^6^

After delivering what for the patient and family are devastating news, it is essential to allow time for the patient (and support persons present), to absorb the information. Silence, although often considered uncomfortable, provides patients with a necessary pause to process and react. Validation of emotions, such as expressing empathy towards a patient’s shock, sadness and anguish, supports emotional health and encourages ongoing honest dialogue. Providing clear recommended next steps and additional resources with rationale and timing, fosters a sense of control for patients. Options may include choices the patient will be asked to make regarding different treatment pathways, palliative care, and other psychological support services. Official guidelines and recommendations should be explained and discussed. Encouraging patients to ask questions in their own time can reduce confusion and enable more active participation in their healthcare decisions, all with the purpose of resulting in improved patient satisfaction and compliance with treatment. If appropriate, open clinical studies pertinent, and especially if recommended, for the patient’s diagnosis and condition should be reviewed, even if such studies are unable to be offered at the clinician’s institution. Also, the clinician should emphasize, that no decision needs to be made at this meeting (if appropriate), and that the patients should fully process the information given, discuss it with trusted support persons, and to offer a follow-up meeting within an appropriate time frame to the situation. It should also be explained, that even if the patient decides for a path that is not considered the ‘standard of care’ it will be respected, with the patient, clinician and the clinicians’ team working together best they can to make such a path successful for the patient. Also, if appropriate, the patient should be given an opportunity to provide their understanding of what the clinician told them prior to concluding the meeting

Finally, offering the follow-up meetings reassures patients that the clinicians support is ongoing. It provides an opportunity for further questions, reflection, and adaptation to the received news. The patient (and support persons) should be encouraged to ask any question on their mind. The possibility for contacting the clinician, and/or the clinicians’ team with questions that the patient subsequently feel cannot wait for the next meeting should be given, with assurance of a no longer than 24h response. Communicating with the clinician and clinician team via the electronic patient portal should be encouraged when appropriate and available.

In most places, an after-visit summary will have been written live by a nurse or another health care professional present at the meeting. In addition, the clinician should provide a concise written summary of what was discussed, what was decided. The level of a patient’s understanding and emotional response, aids continuity of care and ensures that future meetings are informed and patient-centered.^3^ Actually, and with patient consent, involving family members in discussions offers additional emotional support, helps with understanding and aids decision-making, particularly for complex conditions. The presence of family can also provide a buffer against feelings of isolation and despair, facilitating coping for both patients and their loved ones.^7^

Delivering bad news can be emotionally taxing for clinicians, with potential consequences for mental health and burnout. Reflecting on the conversation, seeking peer support, and engaging in debriefs can assist in maintaining emotional well-being and empathy, essential for sustainable compassionate care. As discussed, having a nurse, social worker or similar at the meeting, when possible, not only facilitates patient-provider communication, but may also allow sharing of the emotions generated by the stressful situation.

In summary, in the field of clinical hematology, communicating bad news to patients and their family/trusted friends, requires a nuanced and individual approach that combines clinical expertise with compassionate and comprehensive communication. Adhering to well prepared and thought-out approaches, and providing continuous support, can improve patient outcomes by fostering trust, enhance understanding, and promote meaningful and informed patient decision-making. Adopting such practices can not only facilitate better care for patients but also protect the clinicians’ own well-being, ensuring effective and compassionate health-care professionals. Because malignant hematology is an ever evolving, complex and challenging field, senior experienced hematologists should always include trainees in these situations whenever possible, for the next generation to master these skills, skills that cannot be learned from a textbook, and are likely to be one of the last skills a medical artificial intelligence (AI) ‘clinician’ is able to perform satisfactorily.

## AUTHORSHIP

Writing, editing, conceptualization: Mohamad Mohty Finn Bo Petersen, and Didier Blaise.

## STATEMENTS AND DECLARATIONS

Conceptualization: Mohamad Mohty and Didier Blaise. Writing – original draft: Mohamad Mohty (Lead). Writing – review & editing: Mohamad Mohty, Finn Bo Petersen and Didier Blaise.

## ETHICAL APPROVAL

Not applicable.

## CONSENT TO PARTICIPATE/INFORMED CONSENT

Not applicable.

## CONSENT FOR PUBLICATION

Not applicable.
